# Supplementary material for: Effects of anthropogenic wildfire in low-elevation Pacific island vegetation communities in French Polynesia
Source: PeerJ. 2018 Jun 20;6:e5114. doi: 10.7717/peerj.5114 (PMC6015486; doi:10.7717/peerj.5114)

Supplementary Material

For: EA Newman, CA Winkler, and DH Hembry. Effects of anthropogenic wildfire in low-elevation Pacific island vegetation communities in French Polynesia. PeerJ.

Appendix. Species richness and observed abundances

Figure A1. Species richness (A) and observed abundances of species (B) measured at sampling points in burned and comparison areas at 3 sites. Data represent individuals measured at 13 points each along 5 transects in burned and comparison areas for each of 3 sites (n = 390 data points). Plants are grouped into native, Polynesian introduction, and modern introduction categories.

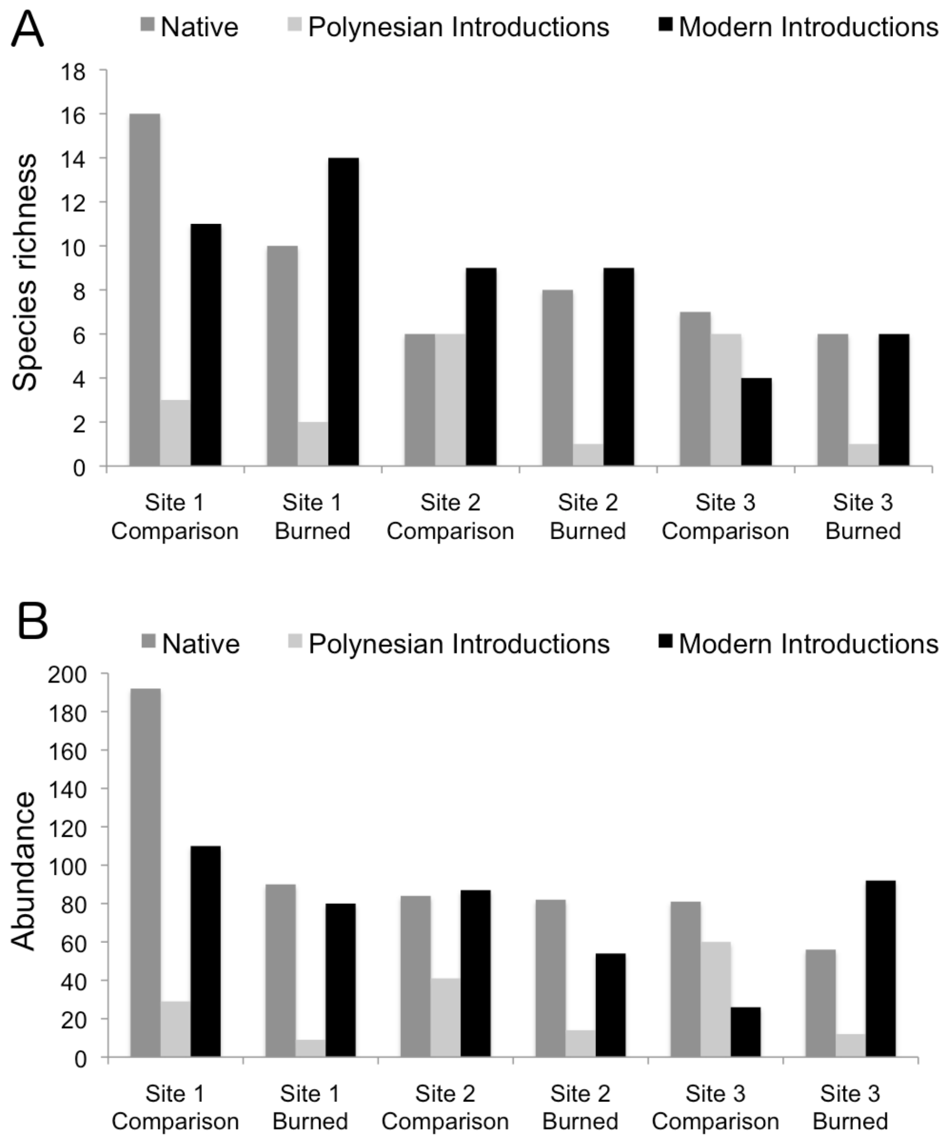

Supplement: Supplemental Information 1 [file peerj-06-5114-s001.pdf]
